# Recent advances in C(sp^3^)–H bond functionalization via metal–carbene insertions

**DOI:** 10.3762/bjoc.12.78

**Published:** 2016-04-25

**Authors:** Bo Wang, Di Qiu, Yan Zhang, Jianbo Wang

**Affiliations:** 1 Beijing National Laboratory of Molecular Sciences (BNLMS), Key Laboratory of Bioorganic Chemistry and Molecular Engineering of Ministry of Education, College of Chemistry, Peking University, Beijing 100871, China

**Keywords:** alkane, diazo compounds, C–H bond functionalization, C–H bond insertion, metal–carbene, site-selectivity

## Abstract

The recent development of intermolecular C–H insertion in the application of C(sp^3^)–H bond functionalizations, especially for light alkanes, is reviewed. The challenging problem of regioselectivity in C–H bond insertions has been tackled by the use of sterically bulky metal catalysts, such as metal porphyrins and silver(I) complexes. In some cases, high regioselectivity and enantioselectivity have been achieved in the C–H bond insertion of small alkanes. This review highlights the most recent accomplishments in this field.

## Introduction

Direct functionalization of inactivated C–H bonds, especially C(sp^3^)–H bonds, have attracted significant attentions in recent years. The C(sp^3^)–H bond activation strategies based on radical reactions and transition metal catalysis have been explored, alongside the development of various directing groups for controlling the site-selectivity of the reaction. Regardless of the great efforts devoted to the field, the intermolecular C(sp^3^)–H bond activation of simple alkanes still remains a formidable challenge, obviously attributed to the inertness and ubiquitous nature of simple aliphatic C(sp^3^)–H bonds. In this context, catalytic metal–carbene C(sp^3^)–H bond insertion represents an alternative and unique approach for this purpose.

**Scheme 1:**
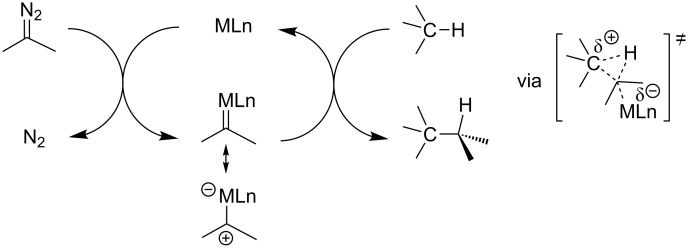
Pathway for transition-metal-catalyzed carbene insertion into C(sp^3^)–H bonds.

Metal–carbene insertion into a C(sp^3^)–H bond, well-recognized as one of the typical reactions of carbene species, have been studied extensively over the decades [1–8]. Mechanistically, the C(sp^3^)–H bond insertion reaction is considered to follow a concerted reaction pathway with a three-center two electron transition state (Scheme 1). Since late transition metals, typically Rh(II) complexes, are most commonly employed as the catalysts, the carbenic carbon of the metal–carbene species is positively charged in general, as shown by the resonance structure. Consequently, when the electron-deficient carbenic carbon approaches the C(sp^3^)–H bonds, the C–H bonds with high electron density will react preferentially [9]. However, the site-selectivity of C(sp^3^)–H bond insertion is also affected by steric factors. High regioselectivity of C(sp^3^)–H bond insertions has been observed in intramolecular reactions in most cases, in which the C(sp^3^)–H bond positioned 5 atoms away from the carbene center will normally react preferentially (1,5 C–H insertion). However, 1,3-, 1,4, and 1,6 C–H insertions are also possible, depending on the substrates and the catalysts. Although the site-selectivity of intramolecular metal–carbene C(sp^3^)–H bond insertion is affected by the combination of factors such as steric and electronic factors as well as catalysts, high site-selectivity is generally achievable, which makes this type of reaction a valuable tool for the construction of carbocycles from readily available starting materials [1–6].

While most of the site-selective metal–carbene C(sp^3^)–H bond insertions are based on intramolecular reaction systems, or with relatively active C–H bonds, such as allylic, benzylic or the C–H bonds adjacent to the oxygen or nitrogen, the corresponding site-selective metal–carbene insertion into simple aliphatic C(sp^3^)–H bonds has also been challenged in intermolecular reaction systems and one has witnessed some exciting advances along this line. Thus, it would be an appropriate time to summarize the field in connection with direct C–H bond functionalization. Since catalytic metal–carbene C(sp^3^)–H bond insertions have been discussed in a series of excellent reviews [1–8], this short article will highlight the most recent developments in the field, with the emphasis on simple aliphatic C(sp^3^)–H bond insertions.

## Review

### Metal carbene C(sp^3^)–H bond insertions into relatively active C–H bonds

Compared to ordinary aliphatic C(sp^3^)–H bonds, the C(sp^3^)–H bonds located at allylic and benzylic sites and those at the α-position of oxygen or nitrogen, show high activity because of the stabilization of the partial positive charge developed in the transition state of the metal–carbene C–H bond insertion process. Such type of intramolecular metal–carbene C–H insertions shows high selectivities in many cases [10–20], and they have been successfully incorporated into the steps in natural product synthesis. Herein some selected recent examples are highlighted.

#### The C–H bond insertions at the α-positions of oxygen or nitrogen

**Scheme 2:**
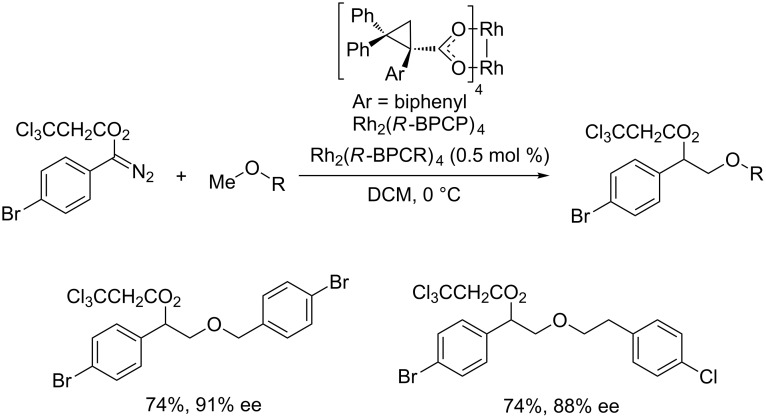
Rh(II)-catalyzed site-selective and enantioselective C–H functionalization of methyl ether.

Attributed to the stabilizing effect of oxygen and nitrogen toward the positive charge development at the neighboring positions, the metal–carbene C–H bond insertions at these positions are favored. Davies and co-workers have recently reported a highly site-selective and enantioselective C–H bond insertion of methyl ethers [21]. The use of 2,2,2-trichloroethyl aryldiazoacetates, in combination with sterically crowded chiral Rh(II) catalysts Rh_2_(*R*-BPCP)_4_, enhances the site-selectivity and the enantioselectivity of the reaction. Interestingly, the C–H bonds of a methyl group show high reactivity over the secondary C–H bonds, even the secondary benzylic C–H bonds (Scheme 2). Notably, for the site-selectivity of carbene insertion into primary, secondary and tertiary C–H bonds, the electronic and steric factors operate in the opposite directions. It is thus possible to tune or even revise the selectivity by judicious combination of reagents and catalysts.

**Scheme 3:**
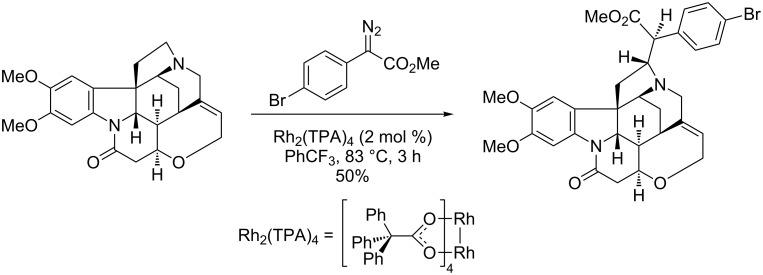
Late-stage C–H functionalization with Rh(II)-catalyzed carbene C(sp^3^)–H insertion.

Rh(II)-catalyzed site-selective and enantioselective intramolecular carbene insertion into the C–H bond at the α-position of a tertiary amine have been previously established by Davies and co-workers [22–24]. Recently, this methodology has been used in the late-stage C–H functionalization of complex alkaloids and drug molecules (Scheme 3) [25].

#### C–H bond insertions at the allylic and benzylic positions

**Scheme 4:**
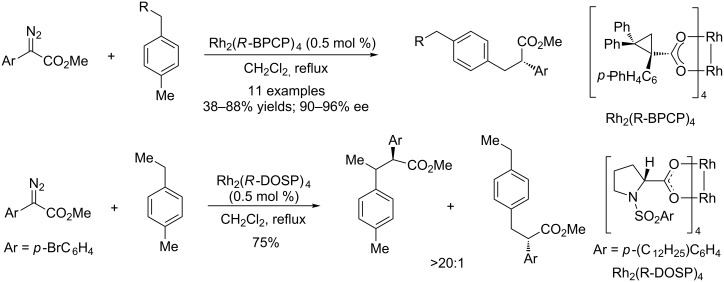
The Rh(II)-catalyzed selective carbene insertion into benzylic C–H bonds.

**Scheme 5:**
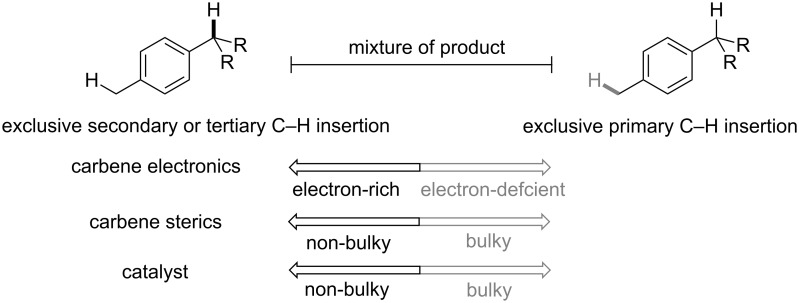
The structure–selectivity relationship.

Metal–carbene C–H insertion is also favored for allylic and benzylic sites. In 2014, Davies and co-workers reported an enantioselective C–H insertion catalyzed by chiral dirhodium catalysts. The reaction took place selectively at the primary C–H bond when chiral Rh(II) catalyst Rh_2_(R-BPCP)_4_ is employed. However, when chiral Rh(II) catalyst Rh_2_(R-DOSP)_4_ is used, secondary C–H bond insertion becomes predominant (Scheme 4) [26]. Subsequently, an extensive structure–selectivity relationship was carried out by Davies and Sigman [27]. The quantitative analysis of the substrate and the reagent shows that a non-bulky electron-rich carbene and a non-bulky catalyst prefer secondary or tertiary C–H bonds, while bulky electron-deficient carbene and bulky catalysts prefer primary C–H bonds (Scheme 5). Such quantitative understanding is very useful for the design of reagent/catalyst combination to achieve high selectivity of unactivated C(sp^3^)–H bond functionalization.

### Metal carbene insertion into aliphatic C(sp^3^)–H bonds of light alkanes

Site-selective metal–carbene insertion into simple aliphatic C(sp^3^)–H bonds is generally more challenging simply because of the ubiquitous nature of C(sp^3^)–H bonds and the little difference between different C(sp^3^)–H bonds. The investigation in this field is focused on the development of novel transition metal catalysts through judicious combination of metal and ligands. In 1982, Callot and Metz reported Rh(III) porphyrins catalyzed intermolecular C–H insertion, which showed an extraordinary selectivity for 1° C–H bonds [28]. Other metal porphyrin complexes, such as Os [29], Fe, Cu, Ag [30], can also serve as catalysts for C–H insertions, although the C–H insertions with these catalysts are generally less efficient.

**Scheme 6:**
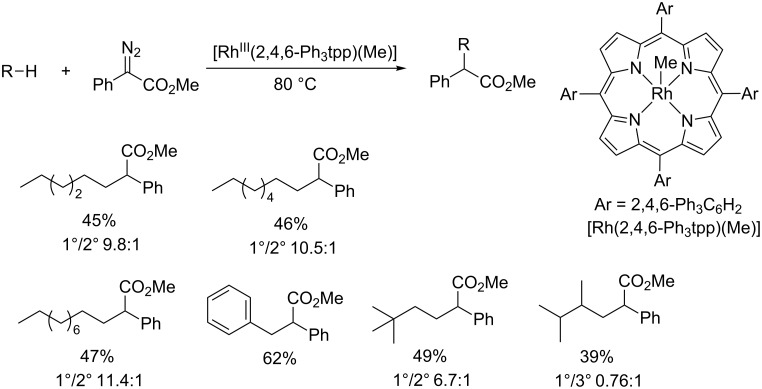
Rh-porphyrin complexes for catalytic intermolecular C–H insertions.

More recently, Che and co-workers developed a highly site-selective intermolecular insertion into primary C–H bonds catalyzed by robust and sterically encumbered [Rh^III^(2,4,6-Ph_3_tpp)Me]. Presumably attributed to the large steric hindrance of 2,4,6-Ph_3_tpp, the reaction for *n*-alkanes afforded a high 1°/2° ratio of up to 11.4:1 (Scheme 6) [31]. The results further demonstrate that steric bulkiness of the catalyst can override the electronic preference for secondary C(sp^3^)–H bond insertions.

**Scheme 7:**
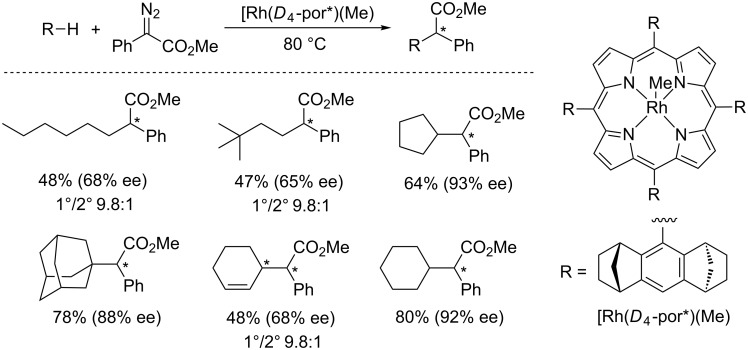
Asymmetric intermolecular C(sp^3^)–H insertion with chiral Rh-porphyrin catalyst.

By employing chiral porphyrins *D*_4_-por*, the enantioselective intermolecular carbene insertion into C(sp^3^)–H bonds was achieved with moderate to high selectivities (Scheme 7). Besides, the reaction of diazo compounds with benzylic, allylic and alkane C(sp^3^)–H bonds afforded the insertion products in up to 80% yield and with up to 93% ee.

**Figure 1:**
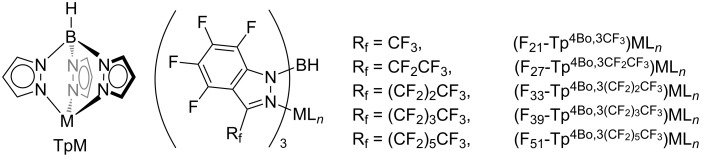
The structure of TpM catalysts.

Pérez and co-workers have explored different types of catalysts based on copper and silver in metal–carbene C(sp^3^)–H insertions. Tris(pyrazoly)borate complexes of copper (TpCu) [32–34] and silver (TpAg) [35–37] have been employed for C(sp^3^)–H insertion of alkanes (Figure 1). Compared to Rh(II) catalysts, these coinage metal-based catalysts are generally less efficient in carbene-transfer reactions, thus requiring a high catalyst loading in general. However, this drawback is compensated by much lower cost of these metals than rhodium.

**Scheme 8:**
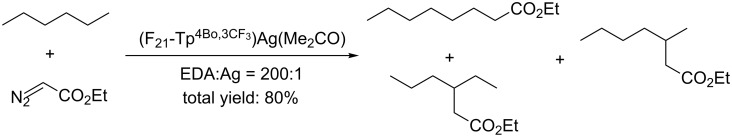
Ag-Tpx-catalyzed intermolecular C–H insertion between EDA and alkanes.

In 2008, Pérez, Díaz-Requejo and Etienne reported a perfluorinated F_21_-tris(pyrazoly)borate (F_21_-Tp) scorpionate ligand, which enhanced alkane C–H functionalization by carbene insertion with (F_21_-Tp)Cu and (F_21_-Tp)Ag catalysts (Figure 1) [38]. In particular, with silver catalyst remarkably low catalyst loading (ca. 0.5%), and high turnover number (up to 200), have been achieved when the reaction was carried out with hexane and EDA (Scheme 8).

The high reactivity of silver-based catalysts encouraged further investigations on its applications in aliphatic C(sp^3^)–H insertions. The metal–carbene C–H bond insertion with methane, the ultimate simple alkane, thus becomes an attractive goal along this line. However, the major obstacle to achieve the methane C–H bond functionalization through metal–carbene C–H insertion is the low reactivity of the methane C–H bond as compared to most of the C–H bonds in organic compounds. This impedes the use of most organic solvents, while the gaseous nature of methane makes it impossible to form a homogenous reaction system without a solvent.

**Scheme 9:**
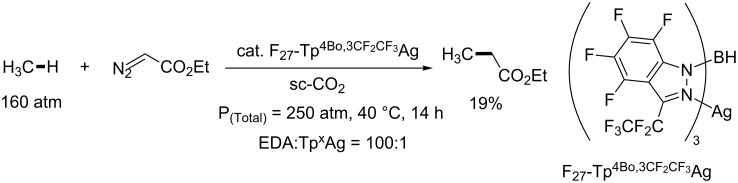
Ag-Tpx-catalyzed C–H insertion of methane with EDA in scCO_2_.

Pérez and co-workers addressed this problem by using supercritical carbon dioxide (scCO_2_) as the reaction medium. Methane can be dissolved in scCO_2_. Moreover, the electron-deficient nature of CO_2_ will prevent its reaction with the highly reactive metal–carbene species, which are electrophilic in nature. In 2011, Pérez, Etienne and Asensio achieved the Tp^x^Ag catalyzed C–H insertion of methane in supercritical CO_2_ (Scheme 9) [39]. The reaction between methane and EDA formed ethyl propionate in 19% yield (based on EDA) at 40 °C over 14 h in the presence of silver-perfluorinated catalysts (F*_n_*-Tp^x^Ag). Other gaseous alkanes, including ethane, propane, butane and isobutene, have also been functionalized with the same catalytic system with EDA [40]. Notably, the site-selectivities of the C–H insertion of pentane in scCO_2_ media are essentially identical as compared to the corresponding reaction with pentane as the solvent.

**Figure 2:**
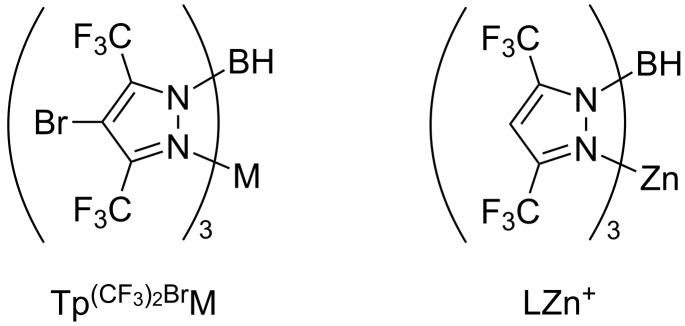
Structure of TpM-type catalysts.

More recently, a new borate ligand, hydrotris((3,5-bis(trifluoromethyl)-4-bromo)pyrazol-1-yl)borate (Tp^(CF3)2,Br^), was introduced and similar C–H bond functionalization of methane was achieved by Tp^(CF3)2,Br^ ML (M = Cu, Ag; L = MeCN or THF) (Figure 2) [41]. A detailed investigation on the TpML-type catalysts was also carried out [42]. Notably, the copper complex Tp^(CF3)2,Br^Cu (NCMe) is soluble in methane/scCO_2_, thus forming a homogenous catalytic system.

**Scheme 10:**
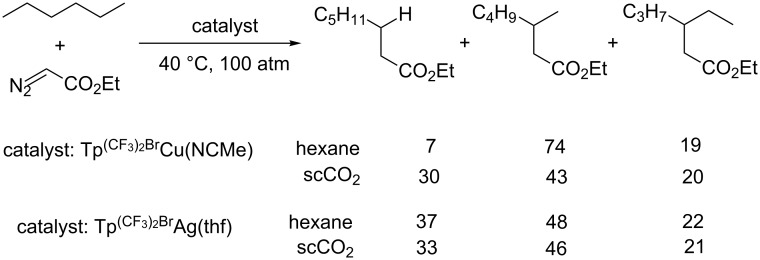
Comparison of site-selectivities of C–H insertion in different reaction media.

With the new borate ligand, depending on the metal of the catalyst, different selectivities were observed when the reaction medium is changed from alkane to scCO_2_ (Scheme 10) [43]. When scCO_2_ was used as the solvent for the C–H insertion of hexane, a significant increase in the functionalization of the primary sites was observed in the presence of Tp^(CF3)2,Br^Cu(MeCN); while hexane and scCO_2_ made little difference as compared to Tp^(CF3)2,Br^Ag(thf) catalyzed reactions. These results may be related to the interaction of the ligand with carbon dioxide. A net electron density flux from the metal center to the ligand and to carbon dioxide was supported by experimental data and DFT studies. Such interaction increases the electrophilicity of the carbene moiety and thus lowers the activation energy for C–H bond insertion.

**Scheme 11:**
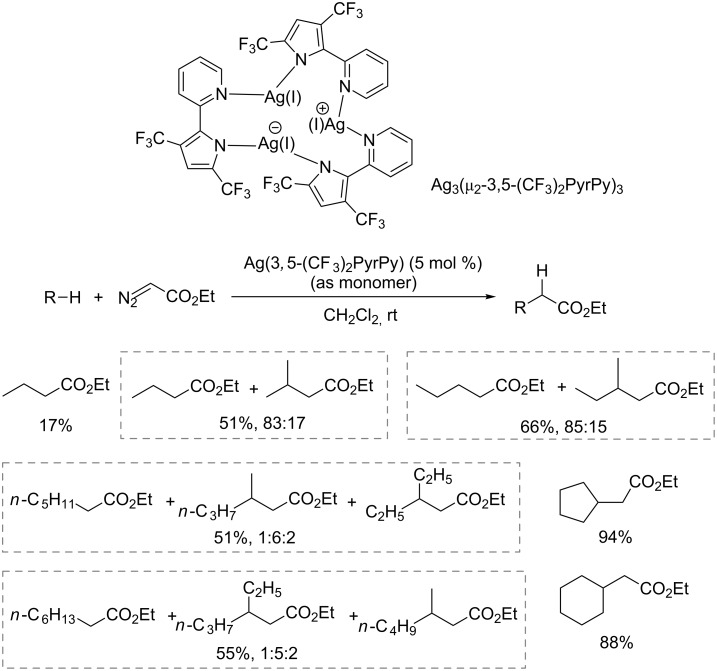
C(sp^3^)–H bond insertion catalyzed by trinuclear cluster Ag.

Other ligands have also been explored for the metal–carbene C(sp^3^)–H insertion of simple alkanes. Mindiola and Caulton reported carbene C–H insertion catalyzed by a trinuclear cluster Ag_3_(μ*_2_*-3,5-(CF_3_)_2_PyrPy)_3_ (3,5-(CF_3_)_2_PyrPy = 3,5-bis(trifluoromethyl)-2,2’-pyridylpyrrolide) [44]. The C–H bond insertion of ethane, propane, butane and more heavier alkanes have been achieved except for methane (Scheme 11). The DFT studies suggested that formation of the silver carbene complex was the rate-determining step for alkane substrates such as ethane and propane. As for methane, the overall rate-determining step was carbene insertion into the C–H bond, attributed to the inertness of the methane C–H bond. As a result, the side reactions of the highly active metallocarbene intermediate override the C–H insertion.

**Scheme 12:**
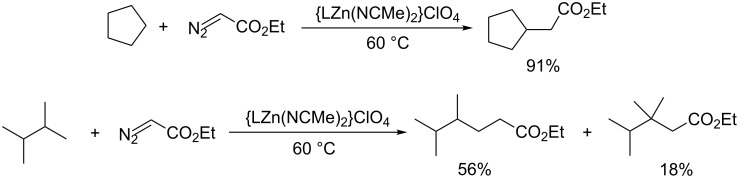
Zn(II)-catalyzed C(sp^3^)–H bond insertion.

Zinc catalysts, which were seldom used for metal–carbene C–H insertions, have been recently explored in the C–H insertion between EDA with alkanes [45]. A detailed computational study of the likely intermediate suggests that it is best described as a zinc-bound carbocation rather than a zinc carbene (Scheme 12). Notably, Zn is a cheap, earth-abundant 3d metal, thus making it attractive as catalyst for C–H bond functionalization.

## Conclusion

The intermolecular metal–carbene insertion represents a direct approach for the C–H bond functionalization of aliphatic C(sp^3^)–H bonds. While some progresses have been made in this area, significant challenge remains. From the selected examples summarized in this review it is obvious that the main issue is the balance of reactivity and selectivity. The factors governing the reactivity and site-selectivity of metal–carbene C–H insertion include: 1) the electrophilicity of the metal–carbene bond; 2) the nucleophilicity of the targeted C–H bond; 3) the steric bulk of the ligand around the metal; 4) the steric bulk of the substituents around the targeted C–H bond. While secondary and tertiary C(sp^3^)–H bonds are favored toward the electron-deficient carbenic carbon center in terms of electronic effects, they are unfavored in terms of steric hindrance. The primary C(sp^3^)–H bonds operate in the opposite way. Further studies in this area will be focused on the development of novel catalysts as well as the perceptive combination of catalyst/substrates in order to achieve highly selective C(sp^3^)–H bond insertions.
